# Innovation in antifungal therapy

**DOI:** 10.1038/s44321-026-00449-x

**Published:** 2026-05-09

**Authors:** Sarah Dellière, Nicolas Papon

**Affiliations:** 1 Université Paris Cité, AP-HP, Parasitology-Mycology Department, Groupe Hospitalier Saint-Louis-Lariboisière-Fernand-Widal, Paris, France; 2 Immunology of Fungal Infection, Institut Pasteur, Université de Paris Cité, Mycology Department, Inserm U1359, Paris, France; 3 Univ Angers, Univ Brest, IRF, SFR ICAT, Angers, France

**Keywords:** Microbiology, Virology & Host Pathogen Interaction, Pharmacology & Drug Discovery

## Abstract

With a steady increase in the number of at-risk patients, invasive fungal infections now represent a significant public health problem. These infections, caused by major pathogens such as *Aspergillus, Candida, Cryptococcus*, and *Pneumocystis*, affect increasingly diverse patient profiles, primarily immunocompromised individuals, with mortality rates often exceeding 50%. Managing these patients remains challenging, as very few systemic antifungals are currently available, compounded by major resistance issues related to the overuse and misuse of these drugs in clinical practice and agriculture. Fortunately, the field of medical mycology has benefited in recent years from major advances in pathogen-directed therapies, including new compounds, repurposing, new formulations, and the identification of new specific fungal targets. Although most immunomodulatory strategies are far from being implemented in clinical practice, recent breakthroughs in translational research have provided unprecedented hope in developing host-directed approaches leveraging cellular aspects and humoral mediators of immunopathogenesis.

## Abstract (web summary)

In this perspective, N. Papon and S. Dellière discuss recent advances in pathogen- and host-directed strategies for future treatment of invasive fungal infections.

## Graphical abstract

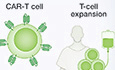

## Introduction

Fungal infections have become a greater global public health concern. Although most superficial fungal infections (skin, nail, hair, mucosae) are rarely fatal, they affect the daily lives of tens of millions of people (e.g. vulvovaginal infections and dermatophytosis). Moreover, the most serious fungal infections impact an ever-growing at-risk population. While chronic or allergic forms are becoming increasingly recognized and diagnosed for people with asthma or chronic obstructive pulmonary disorder (COPD) for instance, invasive fungal infections (IFIs), on the other hand, occur in highly vulnerable patients, such as those with AIDS or cancers, and in transplant recipients. Recent estimates report 6.5 million IFIs and 3.8 million deaths every year, more than a third of which are directly attributable (Denning, 2024).

**Figure 1:**
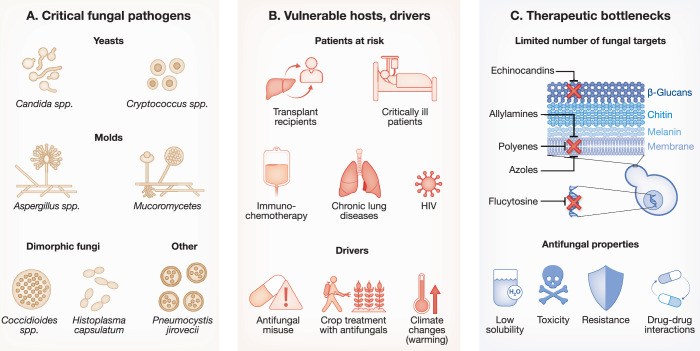
Growing fungal threat and limited therapeutic space. (**A**) Fungal burden. A series of yeast species (e.g.,* Candida* spp. and *Cryptococcus neoformans*), molds (e.g.,* Aspergillus fumigatus* and Mucoromycetes), dimorphic fungi causing endemic mycoses (e.g.,* Histoplasma* spp. and *Coccidioides* spp.), and *Pneumocystis jirovecii* are among the most frequent agents and are now at the top of the WHO priority list of fungal pathogens. (**B**) Growing population of vulnerable individuals and factors promoting the emergence of pathogenic/resistant fungi. Emerging vulnerable patients to invasive fungal infections include transplant recipients, critically ill patients, patients with chronic lung diseases or undergoing immunotherapy, and people infected with HIV. The major drivers of this growing fungal threat are climate change and the antifungal resistance emerging from the misuse of drugs in the clinics and pesticides in agriculture. (**C**) Therapeutic bottlenecks in the management of fungal infections. Currently used antifungals target various fungal biocompounds and cell compartments including ergosterol (polyenes) and its biosynthetic pathway (allylamine, azoles), cell wall biogenesis (echinocandins), and DNA/RNA synthesis (pyrimidine derivatives). Antifungal therapy remains limited by suboptimal pharmacokinetic properties, toxicity, drug–drug interactions, and the accelerating emergence of resistance. Created with Microsoft PowerPoint.

A series of yeast species (e.g.,* Candida albicans, Candida auris*, and *Cryptococcus neoformans*), molds (e.g.,* Aspergillus fumigatus* and Mucoromycetes), dimorphic fungi causing endemic mycoses (e.g.,* Histoplasma* spp.), and *Pneumocystis jirovecii* are among the most frequent agents and are now at the top of the WHO priority list of fungal pathogens (Division, 2022) (Fig. 1A). Additionally, climate changes we are witnessing may have caused a global shift in the epidemiology of fungal infections, notably with the emergence and spread of thermotolerant species (e.g.,* Candida auris, Coccidioides sp*.) that are more likely to infect humans (Case et al, 2025) (Fig. 1B). One of the major challenges in treating life-threatening IFIs stems from the limited availability of antifungal drugs. Currently only five classes of systemic drugs are approved including polyenes (e.g., amphotericin B), azoles (e.g., voriconazole), pyrimidine derivatives (e.g., flucytosine), echinocandins (e.g., caspofungin), and allylamines (e.g., terbinafine), with specific spectrum of activity, pharmacokinetics, and toxicity that must be considered for each use (Fig. 1C). The close evolutionary and cellular similarity between fungi and human hosts limits the availability of selective drug targets. This contributes to host toxicity and complicates antifungal drug discovery. Finally, the misuse and overuse of antifungals in clinics and agriculture have led to the rise of antifungal resistance in many fungal pathogens, posing a significant threat to global health (van Rhijn and Rhodes 2025). It is worth mentioning that recent advances strongly suggest that resistance likely represents the tip of the iceberg, as our understanding of the molecular mechanisms underlying fungal tolerance and heteroresistance remains limited (Zhai et al, 2024; Chen et al, 2023). On this basis, although major advances have been made in the last two decades regarding the diagnosis of mycoses (Salmanton-García et al, 2022), there is still an urgent need for therapeutic innovation for managing deadly IFIs. Here, we summarize the major advances recently made in pathogen-directed therapies including new compounds, repurposing, new formulation, and the identification of new specific fungal targets. We also discuss recent groundbreaking works on host-directed approaches leveraging cellular aspects and humoral mediators of immunopathogenesis.

## Pathogen-directed therapy

The currently used antifungals target diverse fungal biocompounds and cell compartments including ergosterol (polyenes) and its biosynthetic pathway (allylamine, azoles), cell wall biogenesis (echinocandins), and DNA/RNA synthesis (pyrimidine derivatives). Despite their clinical utility, antifungal therapy remains constrained by suboptimal pharmacokinetic properties, toxicity, drug–drug interactions, and the accelerating emergence of resistance, all of which underscore the urgent need for continued innovation in antifungal research (Fisher et al, 2022).

**Figure 2:**
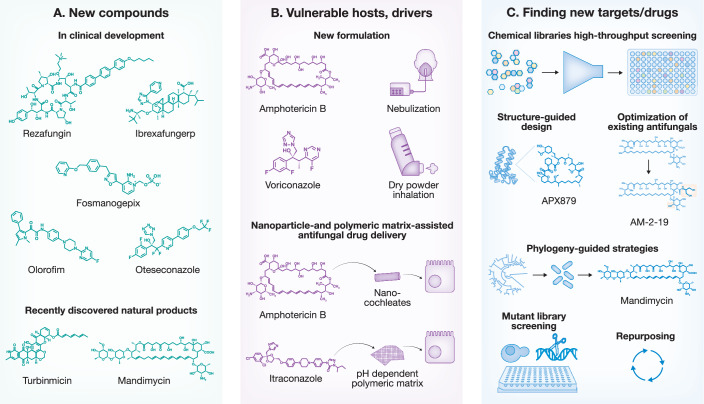
Pathogen-directed therapeutic innovations. (**A**) News drugs. Several next-generation antifungals are advancing through clinical development. These include the second generation echinocandin rezafungin, the triterpenoid ibrexafungerp, and fosmanogepix, i.e. the prodrug of the pyridine-isoxazole manogepix (the three interfering with the fungal cell wall biogenesis), the tetrazole oteseconazole (targeting ergosterol biosynthetic pathway), and the orotomide olorofim (impairing fungal pyrimidine biosynthesis). The most exciting natural products with antifungal activity recently discovered include the marine secondary metabolite turbinmicin disrupting fungal vesicle-mediated trafficking and the polyene mandimycin that specifically targets fungal phospholipids. (**B**) Recent advances in bioengineering for antifungal delivery strategies. In past years, we have witnessed innovations in drug formulation accompanying developments in antifungal therapy. The main hope lies in optimizing currently approved drugs for nebulization administration (e.g., amphotericin B) or dry powder inhalation (e.g., voriconazole) to allow adjunctive local administration leading to higher exposure at the site of infection. Nanoparticle-assisted antifungal drug delivery is also booming and has shown great promise, as exemplified with the encochleated amphotericin B and SUBA-itraconazole. (**C**) Emerging strategies for identifying new fungal targets and antifungals. These include high-throughput screening of structurally diverse chemical libraries, structure-guided design of fungal-selective inhibitors, optimization of the chemical structure of existing antifungals, phylogeny-guided strategies, repurposing and screening of mutant libraries.

In this context, several next-generation antifungals are advancing through clinical development (Fig. 2A). Some represent optimized derivatives of established classes, whereas others introduce new chemical scaffolds and mechanisms of action. Above all, these next-generation antifungals show preferable pharmacokinetics, safety, and efficacy profile, while in some cases overcome existing resistance mechanism. These include the second generation echinocandin rezafungin, the triterpenoid ibrexafungerp, and fosmanogepix, i.e., the prodrug of the pyridine-isoxazole manogepix (the three interfering with the fungal cell wall biogenesis), the tetrazole oteseconazole (targeting ergosterol biosynthetic pathway), and the orotomide olorofim (impairing fungal pyrimidine biosynthesis) (Hoenigl et al, 2024).

Recent advances in bioengineering for antifungal delivery strategies leverage novel targets, including the infectious microenvironment and pathophysiological barriers at infection sites. This is especially relevant in complex cases, such as refractory, recurrent, and drug-resistant invasive fungal infections (Wu et al, 2023). For instance, in past years, we have witnessed innovations in drug formulation accompanying developments in antifungal therapy (Fig. 2B). This is particularly the case for inhaled drugs that deliver the active compound directly to the lungs at convenient concentrations, thereby minimizing deleterious systemic exposure (e.g., drug interactions and toxicities). The main hope lies in optimizing currently approved drugs for nebulization administration (e.g., amphotericin B) or dry powder inhalation (e.g., itraconazole and voriconazole), and developing novel drugs designed specifically for inhalation delivery (e.g., opelconazole). Nanoparticle-assisted antifungal drug delivery is also booming and has shown great promise, as exemplified with the encochleated amphotericin B, corresponding to the historical antifungal trapped in a multilayer nanostructure composed of lipid bilayers and calcium ions (Hoenigl et al, 2024). Finally, such innovations in the formulation of existing antifungals also appear to be effective for oral administration. This is illustrated with SUBA-itraconazole, a combination of the triazole antifungal with a pH-dependent polymeric matrix, that benefits from improved absorption capacity and reduced adverse events compared to conventional itraconazole.

In the coming years, as this has been a success for malaria for instance, we should mention that clinical research might continue for identifying potent combinations of existing antifungals, particularly for treating challenging fungal infections. Pathogen-directed antifungal therapeutic innovations will inevitably involve interdisciplinary strategies aimed at developing new molecules that offer significant PK/PD advantages. However, apart from the recent discovery of marine natural product turbinmicin disrupting fungal vesicle-mediated trafficking, it has become apparent that globally few new antifungal drugs have seen the light of the day in recent years (Zhang et al, 2020). This is mainly due to the small number of known specific fungal targets and the exhaustion of conventional activity-guided drug discovery strategies (Zhang et al, 2020; Papon et al, 2025)

Fortunately, it is highly likely that we will benefit in the short term from major spin-offs in the field of antifungal research thanks to the development of a myriad of powerful integrative approaches (Fig. 2C). These include high-throughput screening of structurally diverse chemical libraries coupled with chemogenomic approaches or structure-guided design of fungal-selective inhibitors that could lead to potent synthetic molecules with either intrinsic antifungal properties or interfere with antifungal mechanisms. This is for instance exemplified by the recent identification of butyrolactol A, an enhancer of caspofungin efficacy that triggers flippase inhibition in drug-resistant fungal pathogens (Chen et al, 2026). Emerging phylogeny-guided strategies have also already led to the discovery of unprecedented natural products with exciting antifungal activities, such as the recently reported polyene mandimycin (Deng et al, 2025). While repurposing—the process of finding new uses for existing medicines—has offered from the past decade, interesting avenues for the development of antimicrobials that are effective against multi-drug-resistant pathogens, new conceptual frameworks seem very promising for optimizing the chemical structure of existing antifungals (Roque-Borda et al, 2025; Maji et al, 2023). Of course, functional genomics based on de novo generation of CRISPR-Cas9 or RNA interference fungal mutant strains or screening of pre-existing mutant libraries remains an efficient method for expanding the therapeutic target space for antifungal development (Santana et al, 2023; Soliman et al, 2021; Middleton et al, 2025).

Finally, although the currently available in vitro and in vivo models for preclinical medical mycology research are relevant for antifungal therapeutic development, the immunological, pharmacological, and anatomical differences between these models and humans remain a major source of bias in cell culture and animal experimentation (Curtis et al, 2025; Last et al, 2021). Studying the fungal microenvironment and evaluating new potent molecules/combinations using simpler, more experimentally controlled systems based on microfluidics or organoid design will certainly offer promising therapeutic avenues for innovation soon (Harding et al, 2025; Wang et al, 2021; Kim et al, 2021). Similarly, current methods used to measure the in vitro activity of antifungal compounds are physiologically implausible in vivo. Therefore, there is room for innovation in this field, for example, in the development of new synthetic media that mimic specific physiological conditions and microenvironments (Vahedi-Shahandashti et al, 2025; Ruhluel et al, 2022).

## Host-directed therapy

IFIs are often described as infections caused by opportunistic pathogens, a view that underestimates the active and dynamic contribution of host immunity to disease development concept (Casadevall and Pirofski, 2003). Fungal pathogenesis does not occur at immune homeostasis. Despite its significant potential, the host immune response has largely been overlooked as a distinguishing target in the pathogenesis of IFIs (Lionakis et al, 2023). In recent years, immunoengineering approaches applied to IFIs, including cell therapies, immunomodulating small molecule delivery, and next generation vaccine development has emerged (Tatara et al, 2025). Although the immune determinants underlying pathogenesis varies across fungal pathogens, strategies can be collectively divided according to their immune targets: (i) phagocyte-mediated, (ii) lymphocyte-mediated, and (iii) humoral-mediated immunity (Manchon et al, 2026).

**Figure 3:**
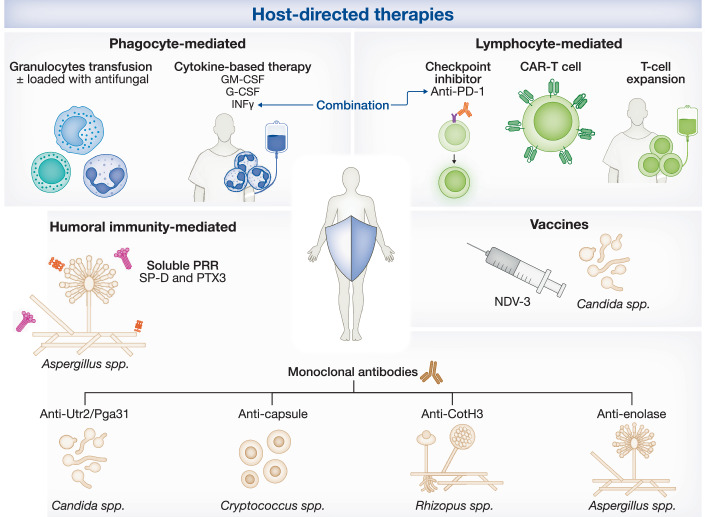
Host-directed therapeutic strategies against fungal infections. Phagocyte-mediated approaches (top left panel). Strategies that aim to enhance innate immune responses include granulocyte transfusions, which can be combined with antifungal loading. This approach is used for severely immunocompromised patients. Cytokine-based therapies, such as GM-CSF, G-CSF and IFNγ, boost phagocyte function. These approaches can be combined with immune checkpoint modulation to further enhance antifungal immunity. Lymphocyte-mediated approaches (top right panel). Strategies for enhancing the adaptive immune system include the use of immune checkpoint inhibitors (e.g., anti-PD-1) to restore T-cell function, adoptive T-cell therapies such as CAR-T cells that target fungal antigens, and the expansion of T-cells ex vivo followed by their reinfusion to improve antifungal responses. Humoral immunity-mediated approaches (left and bottom panels). Antibody-based strategies target key fungal components across major pathogens, including monoclonal antibodies directed against enolase, capsule components, CotH3, or Utr2/Pga31. In parallel, soluble pattern recognition receptors such as SP-D and PTX3 represent promising immunotherapeutic adjuncts by enhancing fungal recognition, inhibition and clearance. Vaccines (right panel). Strategies for preventive and therapeutic vaccination are under development. These include NDV-3 and other candidates that target conserved fungal antigens. The aim is to induce protective immunity against invasive fungal diseases.

Phagocyte cells, primarily neutrophils and macrophages, play a pivotal role in combating molds such as *Aspergillus fumigatus* and Mucorales, as well as yeasts such as *Candida*. Fungal elements are recognized by pattern recognition receptors (PRRs), which trigger the release of cytokines, thereby enhancing phagocytosis and promoting adaptive immune response. Neutrophils generate reactive oxygen species (ROS), which contribute broadly to antifungal activity, and can also release neutrophil extracellular traps (NETs), which are particularly effective against the hyphal fungal morphotype. Cytokine-based therapy using colony-stimulating factors (CSF) has been tried in a small patient cohort with diverse IFIs, suggesting an overall satisfying response rate (Chen et al, 2022) (Fig. 3). A clinical trial showed that administering GM-CSF in allogenic stem cell transplant recipients decreased the incidence of invasive candidiasis and associated mortality (Wan et al, 2015). Interferon-ƴ, which is essential to both innate and adaptive immunity, has been evaluated as an adjunctive therapy in several clinical contexts. It has demonstrated protection against aspergillosis, accelerated fungal clearance in HIV-associated cryptococcosis, and enhanced HLA-DR expression on monocytes in patients with candidemia (Armstrong-James et al, 2017). Cell-based therapies, such as granulocyte transfusion, are characterized by low-certainty data and do not improve mortality rates in cases of invasive aspergillosis. These therapies also carry significant risks (Raad et al, 2013; Sykes et al, 2022).

Defects in T-cell-mediated immunity are well-established risk factors for certain IFIs such as cryptococcosis and *Pneumocystis* pneumonia. However, no T cell-directed immunotherapy has yet progressed beyond the animal model stage for these diseases (Fu et al, 2023). In invasive aspergillosis, lymphocytes appear to be redundant in hosts with an intact myeloid compartment. Nevertheless, adoptive T cell-based strategies, such as expanding naturally occurring antifungal T cells or using engineered approaches like chimeric antigen receptor (CAR) T cells, may provide therapeutic benefit in patients with profound myeloid dysfunction (Seif et al, 2022) (Fig. 3).

There is growing evidence that the immunosuppressive microenvironments observed in IFIs and cancer share key features of immune exhaustion mediated by checkpoint pathways such as PD-1. Program cell death protein 1 is a transmembrane immunoregulatory receptor expressed primarily on the surface of activated T-lymphocytes functioning as an immune checkpoint that negatively regulates immune response. This conceptual overlap has led to the use of immune checkpoint blockade therapy for severe fungal diseases. Anti-PD-1 monoclonal antibodies combined with recombinant IFN-gamma have been successfully used to treat refractory cases of invasive aspergillosis (Serris et al, 2022) and mucormycosis (Mhenni et al, 2024) (Fig. 3). Similar strategies have also demonstrated efficacy for multiple fungal models in preclinical studies (Wurster et al, 2022; Liu et al, 2026), providing a strong rationale for evaluation in randomized controlled trials.

Although often overlooked, humoral immunity plays a central role in host defense against IFIs (Dellière et al, 2020). Soluble mediators, including complement components, acute-phase proteins, collectins, antimicrobial peptides and immunoglobulins orchestrate fungal recognition, opsonization, growth inhibition and the amplification of inflammatory signaling. From a therapeutic standpoint, humoral strategies can be broadly divided into two categories: soluble pattern-recognition receptor (PRR)–based approaches and antibody-based interventions (Fig. 3).

While no soluble PRR-based therapy has yet reached clinical use, several candidates show strong preclinical promise. Surfactant protein D, for example, enhances antifungal immune functions and exerts direct fungistatic activity against *Aspergillus fumigatus*, and has been shown to improve fungal clearance when administered by nebulization in murine models (Dellière and Aimanianda, 2023). Importantly, multiple recombinant humoral mediators are already approved for other indications, making repurposing for fungal diseases both feasible and attractive.

Antibody-based strategies have yielded mixed results thus far. For example, polyclonal intravenous immunoglobulins have failed to prevent fungal infections, and no randomized controlled trials have demonstrated a therapeutic benefit in established IFIs (Mouthon and Lortholary, 2003). Several monoclonal antibodies targeting fungal antigens have shown potent efficacy in preclinical models and are awaiting clinical translation (Whitehead et al, 2024). Those targeting fungal virulence factors are particularly promising (Soliman et al, 2021; Gu et al, 2025). In addition, recent important mechanisms insight linking natural IgG antibodies, innate B1a lymphocytes and neutrophils could inform new strategies (Sarden et al, 2022).

Currently, no vaccine is approved for preventing or treating fungal infections in humans. Nevertheless, encouraging results have emerged from experimental and early clinical studies (Whitehead et al, 2024). A few vaccines have reached human testing. One of them is NDV-3, which contains the *Candida albicans* agglutinin-like sequence 3 protein (Fig. 3). It entered a phase 1b/2a clinical trial and was associated with a reduced rate of recurrent vulvovaginal candidiasis during the 12-month follow-up period (Edwards et al, 2018).

Finally, it is highly possible that interesting host-directed therapeutic perspectives may emerge soon from recent studies emphasizing the importance of immunometabolism in the pathogenesis of IFIs (Weerasinghe et al, 2024). Modulating host responses and the metabolic environment are indeed promising fields of research for developing new therapies and providing alternatives to antifungal drugs for managing long-term at-risk individuals (Gonçalves et al, 2020; Huang et al, 2026; Liu et al, 2026).

## Concluding remarks

Despite significant advancements in antifungal drug discovery, formulation, and delivery, the clinical management of IFIs is still limited by toxicity, drug–drug interactions, poor tissue penetration, and the development of resistance. Even the most innovative pathogen-directed compounds may not overcome these limitations when host immune dysfunction is the primary cause of susceptibility to IFIs. Furthermore, the molecules currently used in therapeutics or under development for IFIs primarily act on fungal targets but above all are perceived as stressors by fungal cells. Due to their intrinsic environmental origin, fungi have evolved extremely efficient genomic plasticity and epigenetic regulation to dynamically and adapt to and escape stress. Therefore, it is becoming increasingly clear that the diversity of molecular mechanisms underlying cue adaptation in fungi will pose a significant challenge to the development of pathogen-directed strategies (Priest et al, 2022; Pérez-Arques et al, 2025).

In turn, host-directed strategies offer a uniquely attractive complement to classical antifungal therapy in this context by restoring or modulating immune effector functions. These strategies have the potential to be less susceptible to resistance, synergize existing antifungals, and limit cumulative toxicity. However, although recent advances in translational research have provided unprecedented hope in this field, most immunomodulatory strategies are far from being implemented in clinical practice. In the future, innovation in therapeutics for IFIs should align and not antagonize innovation in treatment of underlying diseases (e.g., acute leukemias). In this respect, research in therapeutic development for IFIs might be positioned in the context of broader treatment ecosystem. Indeed, host directed defense augmentation may also help control of underlying disease (Pikoulas et al, 2026).

The rarity, heterogeneity, and severity of IFIs make adequately powered randomized controlled trials particularly challenging in mycology, which hinders their evaluation. Nevertheless, a growing body of compelling case series and mechanistic preclinical studies provides a strong rationale to move forward. Progress will require international collaborative trial networks, standardized case definitions, and innovative trial designs adapted to critically ill and immunocompromised populations. Ultimately, integrating host-directed and pathogen-directed therapies is the most promising way to achieve safer, more effective, and more durable antifungal treatment strategies.

## Supplementary information

Peer Review File

## References

[CR1] Armstrong-James D, Brown GD, Netea MG, Zelante T, Gresnigt MS, van deVeerdonk FL, Levitz SM (2017) Immunotherapeutic approaches to treatment of fungal diseases. Lancet Infect Dis 17:e393–e402 28774700 10.1016/S1473-3099(17)30442-5

[CR2] Casadevall A, Pirofski L (2003) The damage-response framework of microbial pathogenesis. Nat Rev Microbiol 1:17–24 15040176 10.1038/nrmicro732 PMC7097162

[CR3] Case NT, Gurr SJ, Fisher MC, Blehert DS, Boone C, Casadevall A, Chowdhary A, Cuomo CA, Currie CR, Denning DW et al (2025) Fungal impacts on Earth’s ecosystems. Nature 638:49–57 39910383 10.1038/s41586-024-08419-4 PMC11970531

[CR4] Chen L, Zhang L, Xie Y, Wang Y, Tian X, Fang W, Xue X, Wang L (2023) Confronting antifungal resistance, tolerance, and persistence: advances in drug target discovery and delivery systems. Adv Drug Deliv Rev 200:115007 37437715 10.1016/j.addr.2023.115007

[CR5] Chen TK, Batra JS, Michalik DE, Casillas J, Patel R, Ruiz ME, Hara H, Patel B, Kadapakkam M, Ch’Ng J et al (2022) Recombinant human granulocyte-macrophage colony-stimulating factor (rhu GM-CSF) as adjuvant therapy for invasive fungal diseases. Open Forum Infect Dis 9:ofac535 36381625 10.1093/ofid/ofac535 PMC9645583

[CR6] Chen X, Duan HD, Hoy MJ, Koteva K, Spitzer M, Guitor AK, Puumala E, Fiebig AA, Hu G, Yiu B et al (2026) Butyrolactol A enhances caspofungin efficacy via flippase inhibition in drug-resistant fungi. Cell 189:620–639.e28 41478284 10.1016/j.cell.2025.11.036 PMC12774453

[CR7] Curtis A, Oladimeji F, Kavanagh K (2025) Models for studying fungal pathogenesis. Curr Clin Microbiol Rep 12:12

[CR8] Dellière S, Aimanianda V (2023) Humoral immunity against *Aspergillus fumigatus*. Mycopathologia 188:603–621 10.1007/s11046-023-00742-0 PMC10249576 37289362

[CR9] Dellière S, Wong SSW, Aimanianda V (2020) Soluble mediators in anti-fungal immunity. Curr Opin Microbiol 58:24–31 32604018 10.1016/j.mib.2020.05.005

[CR10] Deng Q, Li Y, He W, Chen T, Liu N, Ma L, Qiu Z, Shang Z, Wang Z (2025) A polyene macrolide targeting phospholipids in the fungal cell membrane. Nature 640:743–751 40108452 10.1038/s41586-025-08678-9 PMC12003179

[CR11] Denning DW (2024) Global incidence and mortality of severe fungal disease. Lancet Infect Dis 24:e428–e438 10.1016/S1473-3099(23)00692-8 38224705

[CR12] Division WAR (2022) WHO fungal priority pathogens list to guide research, development and public health action. World Health Organization, Geneva

[CR13] Edwards JE Jr, Schwartz MM, Schmidt CS, Sobel JD, Nyirjesy P, Schodel F, Marchus E, Lizakowski M, DeMontigny EA, Hoeg J et al (2018) A fungal immunotherapeutic vaccine (NDV-3A) for treatment of recurrent vulvovaginal candidiasis—a phase 2 randomized, double-blind, placebo-controlled trial. Clin Infect Dis 66:1928–1936 29697768 10.1093/cid/ciy185 PMC5982716

[CR14] Fisher MC, Alastruey-Izquierdo A, Berman J, Bicanic T, Bignell EM, Bowyer P, Bromley M, Brüggemann R, Garber G, Cornely OA et al (2022) Tackling the emerging threat of antifungal resistance to human health. Nat Rev Microbiol 20:557–571 35352028 10.1038/s41579-022-00720-1 PMC8962932

[CR15] Fu MS, Kawakami K, Drummond RA (2023) Antifungal immunity, methods and protocols. Methods Mol Biol 2667:99–112 37145278 10.1007/978-1-0716-3199-7_7

[CR16] Gonçalves SM, Duarte-Oliveira C, Campos CF, Aimanianda V, ter Horst R, Leite L, Mercier T, Pereira P, Fernández-García M, Antunes D et al (2020) Phagosomal removal of fungal melanin reprograms macrophage metabolism to promote antifungal immunity. Nat Commun 11:2282 32385235 10.1038/s41467-020-16120-z PMC7210971

[CR17] Gu Y, Singh S, Alqarihi A, Alkhazraji S, Gebremariam T, Youssef EG, Liu H, Fan X, Jiang W-R, Andes D et al (2025) A humanized antibody against mucormycosis targets angioinvasion and augments the host immune response. Sci Transl Med 17:eads7369 40073153 10.1126/scitranslmed.ads7369 PMC12020122

[CR18] Harding AT, Gehrke L, Vyas JM, Harding HB (2025) Human brain organoids: a new model to study *Cryptococcus neoformans* neurotropism. J Fungi 11:539 10.3390/jof11070539 PMC12295756 40985448

[CR19] Hoenigl M, Arastehfar A, Arendrup MC, Brüggemann R, Carvalho A, Chiller T, Chen S, Egger M, Feys S, Gangneux J-P et al (2024) Novel antifungals and treatment approaches to tackle resistance and improve outcomes of invasive fungal disease. Clin Microbiol Rev 37:e0007423 10.1128/cmr.00074-23 PMC11237431 38602408

[CR20] Huang J, Wang Y, Li F, Zhang N, Tian G, Guo D, Guo Y, Liu Z, Wu Y, Li X et al (2026) Scap stabilizes PKM2 to promote glycolysis and enhance anti-fungal immunity in macrophages. Cell Rep 45:117106 41831231 10.1016/j.celrep.2026.117106

[CR21] Kim J, Lee K-T, Lee JS, Shin J, Cui B, Yang K, Choi YS, Choi N, Lee SH, Lee J-H et al (2021) Fungal brain infection modelled in a human-neurovascular-unit-on-a-chip with a functional blood–brain barrier. Nat Biomed Eng 5:830–846 34127820 10.1038/s41551-021-00743-8

[CR22] Last A, Maurer M, Mosig AS, Gresnigt MS, Hube B (2021) In vitro infection models to study fungal–host interactions. FEMS Microbiol Rev 45:fuab005 33524102 10.1093/femsre/fuab005 PMC8498566

[CR23] Lionakis MS, Drummond RA, Hohl TM (2023) Immune responses to human fungal pathogens and therapeutic prospects. Nat Rev Immunol 23:433–452 36600071 10.1038/s41577-022-00826-w PMC9812358

[CR24] Liu J, Ding H, Tan W, Yu R, Li Y, Liu Y, Liu M, Zhao P, Liu Y, Xu F et al (2026) Meteorin-like is associated with poor outcome in invasive candidiasis in mouse models and in humans. Sci Transl Med 18:eadw8481 41604463 10.1126/scitranslmed.adw8481

[CR25] Maji A, Soutar CP, Zhang J, Lewandowska A, Uno BE, Yan S, Shelke Y, Murhade G, Nimerovsky E, Borcik CG et al (2023) Tuning sterol extraction kinetics yields a renal-sparing polyene antifungal. Nature 623:1079–1085 10.1038/s41586-023-06710-4 PMC10883201 37938782

[CR26] Manchon R, Feys S, Hoenigl M, van de Veerdonk FL, Lanternier F, Wauters J, Carvalho A, Serris A, Dellière S (2026) Aspergillus and host-pathogen interaction: focus on treatment-relevant aspects. Clin Microbiol Infect 32:729–739 10.1016/j.cmi.2025.08.030 40912458

[CR27] Mhenni R, Dellière S, Maaouia CB, Hamane S, Deniau B, Mahévas T, Chaussard M, Coutrot M, Guillemet L, Cupaciu A et al (2024) Combined antifungal therapy with immunostimulation for refractory cutaneous and peritoneal mucormycosis caused by *Rhizopus microsporus*. Diagn Microbiol Infect Dis 111:116653 39689401 10.1016/j.diagmicrobio.2024.116653

[CR28] Middleton G, Mahamud FO, Storer ISR, Williams-Gunn A, Wostear F, Abdolrasouli A, Barclay E, Bradford A, Steward O, Schelenz S et al (2025) Evidence that G-quadruplexes form in pathogenic fungi and represent promising antifungal targets. EMBO Mol Med 17:3636–3656 41249737 10.1038/s44321-025-00340-1 PMC12686049

[CR29] Mouthon L, Lortholary O (2003) Intravenous immunoglobulins in infectious diseases: where do we stand? Clin Microbiol Infect 9:333–338 12848745 10.1046/j.1469-0691.2003.00694.x

[CR30] Papon N, Courdavault V, Chaturvedi V (2025) Phylogeny-guided discovery of new antifungals. Trends Pharm Sci 46:483–485 40374416 10.1016/j.tips.2025.04.004

[CR31] Pérez-Arques C, Navarro-Mendoza MI, Xu Z, Walther G, Heitman J (2025) RNAi epimutations conferring antifungal drug resistance are inheritable. Nat Commun 16:7293 40775213 10.1038/s41467-025-62572-6 PMC12332000

[CR32] Pikoulas A, Morianos I, Nidris V, Hamdy R, Intze E, López-López Á, Moran-Garrido M, Muthu V, Halabalaki M, Papaioanou V et al (2026) Albumin orchestrates a natural host defence mechanism against mucormycosis. Nature 649:693–702 41501454 10.1038/s41586-025-09882-3 PMC12804082

[CR33] Priest SJ, Yadav V, Roth C, Dahlmann TA, Kück U, Magwene PM, Heitman J (2022) Uncontrolled transposition following RNAi loss causes hypermutation and antifungal drug resistance in clinical isolates of *Cryptococcus neoformans*. Nat Microbiol 7:1239–1251 35918426 10.1038/s41564-022-01183-z PMC10840647

[CR34] Raad II, Chaftari AM, Shuaibi MMA, Jiang Y, Shomali W, Cortes JE, Lichtiger B, Hachem RY (2013) Granulocyte transfusions in hematologic malignancy patients with invasive pulmonary aspergillosis: outcomes and complications. Ann Oncol 24:1873–1879 23519997 10.1093/annonc/mdt110 PMC4990830

[CR35] Roque-Borda CA, Medina-Alarcón KP, Pereira JPSG, dos A Sevilhano TC, Aguilar-Morón B, Díaz-Cárdenas F, da Cruz LS, Xavier-Júnior FH, Vicente EF, Perdigão J et al (2025) Repositioning antimicrobial peptides against WHO-priority fungi. Adv Sci 12:e09567 10.1002/advs.202509567 PMC12499507 40884276

[CR36] Ruhluel D, O’Brien S, Fothergill JL, Neill DR (2022) Development of liquid culture media mimicking the conditions of sinuses and lungs in cystic fibrosis and health. F1000Research 11:1007 36519007 10.12688/f1000research.125074.1 PMC9718992

[CR37] Salmanton-García J, Hoenigl M, Gangneux J-P, Segal E, Alastruey-Izquierdo A, Akdagli SA, Lagrou K, Özenci V, Vena A, Cornely OA (2022) The current state of laboratory mycology and access to antifungal treatment in Europe: a European Confederation of Medical Mycology survey. Lancet Microbe 4:e47–e56 10.1016/S2666-5247(22)00261-0 36463916

[CR38] Santana DJ, Anku JAE, Zhao G, Zarnowski R, Johnson CJ, Hautau H, Visser ND, Ibrahim AS, Andes D, Nett JE et al (2023) A *Candida auris*-specific adhesin, Scf1, governs surface association, colonization, and virulence. Science 381:1461–1467 37769084 10.1126/science.adf8972 PMC11235122

[CR39] Sarden N, Sinha S, Potts KG, Pernet E, Hiroki CH, Hassanabad MF, Nguyen AP, Lou Y, Farias R, Winston BW et al (2022) A B1a–natural IgG–neutrophil axis is impaired in viral- and steroid-associated aspergillosis. Sci Transl Med 14:eabq6682 36475902 10.1126/scitranslmed.abq6682

[CR40] Seif M, Kakoschke TK, Ebel F, Bellet MM, Trinks N, Renga G, Pariano M, Romani L, Tappe B, Espie D et al (2022) CAR T cells targeting *Aspergillus fumigatus* are effective at treating invasive pulmonary aspergillosis in preclinical models. Sci Transl Med 14:eabh1209 36170447 10.1126/scitranslmed.abh1209

[CR41] Serris A, Ouedrani A, Uhel F, Gazzano M, Bedarida V, Rouzaud C, Bougnoux M-E, Raphalen J-H, Poirée S, Lambotte O et al (2022) Case Report: immune checkpoint blockade plus interferon-Γ add-on antifungal therapy in the treatment of refractory covid-associated pulmonary aspergillosis and cerebral mucormycosis. Front Immunol 13:900522 35720319 10.3389/fimmu.2022.900522 PMC9199385

[CR42] Soliman SSM, Baldin C, Gu Y, Singh S, Gebremariam T, Swidergall M, Alqarihi A, Youssef EG, Alkhazraji S, Pikoulas A et al (2021) Mucoricin is a ricin-like toxin that is critical for the pathogenesis of mucormycosis. Nat Microbiol 6:313–326 33462434 10.1038/s41564-020-00837-0 PMC7914224

[CR43] Sykes DB, Martinelli MM, Negoro P, Xu S, Maxcy K, Timmer K, Viens AL, Alexander NJ, Atallah J, Snarr BD et al (2022) Transfusable neutrophil progenitors as cellular therapy for the prevention of invasive fungal infections. J Leukoc Biol 111:1133–1145 35355310 10.1002/JLB.4HI1221-722R PMC9133213

[CR44] Tatara AM, Mikos AG, Kontoyiannis DP (2025) Immunoengineering: an emerging field in infectious diseases. J Infect Dis 232:28–35 40259762 10.1093/infdis/jiaf209 PMC12308683

[CR45] Vahedi-Shahandashti R, Hofer I, Leighton H, Fothergill JL, Neill DR, Lass-Flörl C (2025) The impact of respiratory tract mimicking media on antifungal susceptibility testing. Antimicrob Agents Chemother 69:e00225 40525409 10.1128/aac.00225-25 PMC12327005

[CR46] van Rhijn N, Rhodes J (2025) Evolution of antifungal resistance in the environment. Nat Microbiol 10:1804–1815 40730910 10.1038/s41564-025-02055-y

[CR47] Wan L, Zhang Y, Lai Y, Jiang M, Song Y, Zhou J, Zhang Z, Duan X, Fu Y, Liao L et al (2015) Effect of granulocyte-macrophage colony-stimulating factor on prevention and treatment of invasive fungal disease in recipients of allogeneic stem-cell transplantation: a prospective multicenter randomized phase IV trial. J Clin Oncol 33:3999–4006 26392095 10.1200/JCO.2014.60.5121

[CR48] Wang X, Wang S, Guo B, Su Y, Tan Z, Chang M, Diao J, Zhao Y, Wang Y (2021) Human primary epidermal organoids enable modeling of dermatophyte infections. Cell Death Dis 12:35 33414472 10.1038/s41419-020-03330-y PMC7790817

[CR49] Weerasinghe H, Stölting H, Rose AJ, Traven A (2024) Metabolic homeostasis in fungal infections from the perspective of pathogens, immune cells, and whole-body systems. Microbiol Mol Biol Rev 88:e00171–22 39230301 10.1128/mmbr.00171-22 PMC11426019

[CR50] Whitehead AJ, Woodring T, Klein BS (2024) Immunity to fungi and vaccine considerations. Cell Host Microbe 32:1681–1690 39389032 10.1016/j.chom.2024.09.011 PMC11980782

[CR51] Wu S, Song R, Liu T, Li C (2023) Antifungal therapy: novel drug delivery strategies driven by new targets. Adv Drug Deliv Rev 199:114967 37336246 10.1016/j.addr.2023.114967

[CR52] Wurster S, Watowich SS, Kontoyiannis DP (2022) Checkpoint inhibitors as immunotherapy for fungal infections: promises, challenges, and unanswered questions. Front Immunol 13:1018202 36389687 10.3389/fimmu.2022.1018202 PMC9640966

[CR53] Zhai B, Liao C, Jaggavarapu S, Tang Y, Rolling T, Ning Y, Sun T, Bergin SA, Gjonbalaj M, Miranda E et al (2024) Antifungal heteroresistance causes prophylaxis failure and facilitates breakthrough *Candida parapsilosis* infections. Nat Med 30:3163–3172 10.1038/s41591-024-03183-4 PMC11840754 39095599

[CR54] Zhang F, Zhao M, Braun DR, Ericksen SS, Piotrowski JS, Nelson J, Peng J, Ananiev GE, Chanana S, Barns K et al (2020) A marine microbiome antifungal targets urgent-threat drug-resistant fungi. Science 370:974–978 33214279 10.1126/science.abd6919 PMC7756952

