# Supplementary material for: Innovation in antifungal therapy
Source: EMBO Mol Med. 2026 May 9;18(7):2523–9. doi: 10.1038/s44321-026-00449-x (PMC13365550; doi:10.1038/s44321-026-00449-x)
Supplement: Supplementary file 1 — Peer Review File [file 44321_2026_449_MOESM1_ESM.pdf]

EMBO Molecular Medicine

# Innovation in antifungal therapy

Dellière Sarah and Nicolas Papon

*Corresponding author: Nicolas Papon (nicolas.papon@univ-angers.fr), Dellière Sarah (sarah.delliere@aphp.fr)*



## Review Timeline:

| | |
|---|---|
| Submission Date: | 11th Feb 26 |
| Editorial Decision: | 10th Mar 26 |
| Revision Received: | 7th Apr 26 |
| Accepted: | 13th Apr 26 |

*Editor: Zeljko Durdevic*

## Transaction Report:

(Note: With the exception of the correction of typographical or spelling errors that could be a source of ambiguity, letters and reports are not edited. Depending on transfer agreements, referee reports obtained elsewhere may or may not be included in this compilation. Referee reports are anonymous unless the Referee chooses to sign their reports.)

10th Mar 2026

Dear Prof. Papon,

Thank you for the submission of your manuscript to EMBO Molecular Medicine. We have now received feedback from the two reviewers who agreed to evaluate your manuscript. As you will see from the reports below, both referees are positive about its interest and timeliness, however, referee #1 raises important criticisms that should be fully addressed in a revised manuscript. Please note that due to the number of references, length of the manuscript text and considering the referees' feedback your manuscript will be published as a Perspective article. Please check our Author Guidelines for more information.
https://link.springer.com/journal/44321/submission-guidelines#cms-Article-types

I would also like to ask you to amend the following:
1) Please revise the figure in response to the referee #1 comment (e.g. consider splitting it into two figures and incorporating original conceptual elements to better distinguish it from prior reviews). Review your figures and figure legends carefully. We will send the article to our graphic artist who will re-draw the figures for style and clarity. Please submit your revised figures in an editable format (e.g. PDF with the option 'editable labels' checked).
2) Please rename "Declaration of interests" to "Disclosure and competing interests statement". We updated our journal's competing interests policy in January 2022 and request authors to consider both actual and perceived competing interests. Please review the policy https://www.embopress.org/competing-interests and update your competing interests if necessary.
3) Author contributions: Please remove it from the manuscript and specify author contributions in our submission system. CRediT has replaced the traditional author contributions section because it offers a systematic machine-readable author contributions format that allows for more effective research assessment. You are encouraged to use the free text boxes beneath each contributing author's name to add specific details on the author's contribution. More information is available in our guide to authors:
https://www.embopress.org/page/journal/17574684/authorguide#authorshipguidelines
4) Funding: Please make sure all funding sources are entered in both the Acknowledgements and in our submission system.
5) As part of the EMBO Publications transparent editorial process EMBO Molecular Medicine will publish online a Review Process File (RPF) to accompany accepted manuscripts. This file will be published in conjunction with your paper and will include the anonymous referee reports, your point-by-point response and all pertinent correspondence relating to the manuscript. Let us know whether you agree with the publication of the RPF.
6) Please provide a point-by-point letter INCLUDING my comments as well as the reviewer's reports and your detailed responses (as Word file).

I hope that the referees' comments do not prove too problematic to address and I look forward to reading your next

version.

Yours sincerely,
Zeljko Durdevic

Zeljko Durdevic
Senior Editor
EMBO Molecular Medicine

*** IMPORTANT INFORMATION ***

When submitting your revised manuscript, please include:

1) a .doc formatted version of the manuscript text (including Figure legends and tables)
2) Separate figure files
3) a letter INCLUDING the reviewer's reports and your detailed responses to their comments.

Also, and to save some time should your paper be accepted, please read below for additional information regarding some features of our research articles:

1) Disclosure and competing interest statement: Please include a statement declaring any competing commercial interests in relation to your submitted work.

2) Please note that we now mandate that all corresponding authors list an ORCID digital identifier. This takes <90 seconds to complete. We encourage all authors to supply an ORCID identifier, which will be linked to their name for unambiguous name identification.

Currently, our records indicate that the ORCID for your account is 0000-0001-6265-7321.

Please click the link below to modify this ORCID:
Link Not Available
-

Thank you,

Zeljko Durdevic

Zeljko Durdevic
Editor
EMBO Molecular Medicine

***** Reviewer's comments *****

Referee #1 (Remarks for Author):

Comments to authors

It is a succinct and well readable review that addresss many but not all modes of innovation in modern mycology. Some areas the authors could consider expanding and commenting more are:
A) Bioengineering and novel ways to deliver antifungals
B) Emerging areas such as immunoengineering (see Tatara A et al. JID 2025)
C) Emphasize more the importance of immunometabolism to pathogenesis
D) Monoclonal antibodies against virulence factors (eg Mab against Mucoricin) could be another direction?
E) Discuss more the enormous genomic plasticity and epigenetic regulation of fungi as a challenge to pathogen-directed therapy
F) Position innovation in mycology in the context of innovation in treatment of underlying diseases (e.g. acute leukemias). Innovation in mycology should "align" and not antagonize in the context of broader treatment ecosystem (. Eg host directed immune augmentation to also help immune control of underlying disease
G) Would you like to discuss that resistance is the tip of the iceberg and we do not know much regarding tolerance and heteroresistance?
H) Figure is kind of simplistic and tries to say too much in each panel and similar conceptually to other such figures in prior reviews.
I) How current animal models help or not study fungal disease and therapy, in view of immunologic, pharmacologic and anatomic difference between mice and man? Is there a room of innovation by studying fungal microenvironment with simpler more experimentally controlled systems? (e.g. organoids)
J) Is the way we measure susceptibility/resistance in vitro physiologically plausible to the in vivo situation? Is there room for innovation here? (e.g. studying different media in terms of iron, glucose sources, 02 tension) -

Referee #2 (Remarks for Author):

This is a very nice overview on the innovation of antifungal therapies. The authors have chosen to talk about both pathogen- and host-driven therapies. The paper is very well structured and written, giving the reader an immersive overview on the gaps and developments of much needed antifungal therapy. I just have a few of suggestions to improve an already excellent manuscript:

1- Line 52 - remove "the" climate changes
2- Line 53 - examples of thermotolerant species.
3- Line 138 - ROS are not exclusively targeting hyphal growth.

Dear Editor,

We are grateful for your editorial review of our manuscript.
The reviewers' positive response to our manuscript is appreciated, and we thank you for their insightful suggestions and constructive criticism.
We have addressed all concerns raised in blue in the corrected version as carefully as we can.
We hope that the revised manuscript can be published in EMBO Molecular Medicine.
Please contact us if you have any further questions.
Thank you very much for your time and consideration.

---

## Editorial comments :

Dear Prof. Papon,

Thank you for the submission of your manuscript to EMBO Molecular Medicine. We have now received feedback from the two reviewers who agreed to evaluate your manuscript. As you will see from the reports below, both referees are positive about its interest and timeliness, however, referee #1 raises important criticisms that should be fully addressed in a revised manuscript. Please note that due to the number of references, length of the manuscript text and considering the referees' feedback your manuscript will be published as a Perspective article. Please check our Author Guidelines for more information. https://link.springer.com/journal/44321/submission-guidelines#cms-Article-types

REPLY : In response to reviewers' feedback, we have added sentences, paragraphs, references and two figures to our manuscript. We now agree that the 'Perspectives' format is preferable. Furthermore, we acknowledge that we have favoured and adopted a more 'perspective' tone than a simple commentary overall.

I would also like to ask you to amend the following:

1) Please revise the figure in response to the referee #1 comment (e.g. consider splitting it into two figures and incorporating original conceptual elements to better distinguish it from prior reviews). Review your figures and figure legends carefully. We will send the article to our graphic artist who will re-draw the figures for style and clarity. Please submit your revised figures in an editable format (e.g. PDF with the option 'editable labels' checked).

REPLY : In response to Reviewer 1, we have created three new figures to illustrate the majority of the subsections in our manuscript.

2) Please rename "Declaration of interests" to "Disclosure and competing interests statement". We updated our journal's competing interests policy in January 2022 and request authors to consider both actual and perceived competing interests. Please review the policy https://www.embopress.org/competing-interests and update your competing interests if necessary.

REPLY : Done

3) Author contributions: Please remove it from the manuscript and specify author contributions in our submission system. CRediT has replaced the traditional author contributions section because it offers a systematic machine-readable author contributions format that allows for more effective research assessment. You are encouraged to use the free text boxes beneath each contributing author's name to add specific details on the author's contribution. More information is available in our guide to authors:
https://www.embopress.org/page/journal/17574684/authorguide#authorshipguidelines

REPLY : Done

4) Funding: Please make sure all funding sources are entered in both the Acknowledgements and in our submission system.

REPLY : Done

5) As part of the EMBO Publications transparent editorial process EMBO Molecular Medicine will publish online a Review Process File (RPF) to accompany accepted manuscripts. This file will be published in conjunction with your paper and will include the anonymous referee reports, your point-by-point response and all pertinent correspondence relating to the manuscript. Let us know whether you agree with the publication of the RPF.

REPLY : Yes, we agree.

6) Please provide a point-by-point letter INCLUDING my comments as well as the reviewer's reports and your detailed responses (as Word file). I hope that the referees' comments do not prove too problematic to address and I look forward to reading your next version.

REPLY : Overall, we found the referees’ comments constructive and helpful in improving the manuscript. We have carefully addressed these comments in the revised version. In particular, we have expanded the sections highlighted

by Reviewer 1. However, we have only made limited additions in order to preserve the 'Perspective' nature of the manuscript and avoid exceeding the word count. We hope that the revised version meets your expectations, and we would like to thank you again for the opportunity to improve our work.

## ***** Reviewer's comments *****

**Referee #1 (Remarks for Author):**

It is a succinct and well readable review that addresss many but not all modes of innovation in modern mycology.

REPLY : This positive comment on our manuscript is appreciated.

Some areas the authors could consider expanding and commenting more are:

A) Bioengineering and novel ways to deliver antifungals

REPLY : Version 1 of our manuscript described the most significant advances in bioengineering applied to antifungal therapy innovation (lines 91–102 of version 2). To clarify this section, we have begun the paragraph with a few new sentences and included an excellent recent review of the subject (lines 88-92) : « **Recent advances in bioengineering for antifungal delivery strategies leverage novel targets, including the infectious microenvironment and pathophysiological barriers at infection sites. This is especially relevant in complex cases, such as refractory, recurrent, and drug-resistant invasive fungal infections (Wu et al, 2023). For instance, in past years, we have witnessed innovations in drug formulation accompanying developments in antifungal therapy.** »

B) Emerging areas such as immunoengineering (see Tatara A et al. JID 2025)

REPLY : The second part of version 1 of our manuscript summarises recent advances in the field of immunoengineering. To provide a clearer introduction to this novel area of medical mycology, we have added a new sentence at the beginning of the section 'Host-directed therapy' and included an excellent recent review of the subject (lines 146-148) **: « In recent years, immunoengineering approaches applied to IFIs, including cell therapies, immunomodulating small molecule delivery, and next generation vaccine development has emerged (Tatara et al, 2025). »**

C) Emphasize more the importance of immunometabolism to pathogenesis

REPLY : Although no formal applications have yet emerged from this field, we agree that it is worthwhile to discuss the potential of recent immunometabolism studies in the context of the pathophysiology of fungal infections. To this end, we have added some sentences and references (lines 210-214). **« Finally, it is highly possible that interesting host-directed therapeutic perspectives may emerge soon from recent studies emphasizing the importance of immunometabolism in the pathogenesis of IFIs (Weerasinghe et al, 2024). Modulating host responses and the metabolic environment are indeed promising fields of research for developing new therapies and providing alternatives to antifungal drugs for managing long-term at-risk individuals (Gonçalves et al, 2020; Huang et al, 2026; Liu et al, 2026). »**

D) Monoclonal antibodies against virulence factors (eg Mab against Mucoricin) could be another direction?

REPLY : The reviewer is right. To this end, we have included in the new version (lines 199-202) : **« Several monoclonal antibodies targeting fungal antigens have shown potent efficacy in preclinical models and are awaiting clinical translation (Whitehead et al, 2024). Those targeting fungal virulence factors are particularly promising (Soliman et al, 2021; Gu et al, 2025). »**

E) Discuss more the enormous genomic plasticity and epigenetic regulation of fungi as a challenge to pathogen-directed therapy

REPLY : We also agree that this specific trait of the fungal kingdom is worth discussing in the context of the challenges it poses for the development of pathogen-directed therapies. We have therefore added the following sentences and relevant references to the 'Concluding Remarks' section (lines 221-227) : **« Furthermore, the molecules currently used in therapeutics or under development for IFIs primarily act on fungal targets but above all are perceived as stressors by fungal cells. Due to their primarily environmental origin, fungi have evolved extremely efficient genomic plasticity and epigenetic regulation to dynamically and adapt to and escape stress. Therefore, it is becoming increasingly clear that the diversity of molecular mechanisms underlying cue adaptation in fungi will pose a significant challenge to the development of pathogen-directed strategies (Priest et al, 2022; Pérez-Arques et al, 2025). »**

F) Position innovation in mycology in the context of innovation in treatment of underlying diseases (e.g. acute leukemias). Innovation in mycology should "align" and not antagonize in the context of broader treatment ecosystem (. Eg host directed immune augmentation to also help immune control of underlying disease

REPLY : The reviewer is correct. It is worth reminding ourselves that, in the future, innovation in therapeutics for IFIs should be aligned with, rather than in opposition to, innovation in the treatment of underlying diseases. Research into therapeutic development for IFIs should be positioned within the context of the broader treatment ecosystem. We have now added the following sentences and an excellent recent reference to illustrate this point (lines 232-236) : **« In the future, innovation in therapeutics for IFIs should align and not antagonize innovation in treatment of underlying diseases (e.g. acute leukemias). In this respect, research in therapeutic development for IFIs might be positioned in the context of broader treatment ecosystem. Indeed, host directed defense augmentation may also help control of underlying disease (Pikoulas et al, 2026). »**

G) Would you like to discuss that resistance is the tip of the iceberg and we do not know much regarding tolerance and heteroresistance?

REPLY : Of course, it is also worth reminding readers that resistance is just the tip of the iceberg, and that much remains unknown about tolerance and heteroresistance. To this end, we have added a specific sentence and a couple of references (lines 62-65) : **« It is worth mentioning that recent advances strongly suggest that resistance likely represents the tip of the iceberg, as our understanding of the molecular mechanisms underlying fungal tolerance and heteroresistance remains limited (Zhai et al, 2024; Chen et al, 2023). »**

H) Figure is kind of simplistic and tries to say too much in each panel and similar conceptually to other such figures in prior reviews.

REPLY : Reviewer 1 is right, we have created three new figures to broadly illustrate the majority of the subsections in our manuscript.

I) How current animal models help or not study fungal disease and therapy, in view of immunologic, pharmacologic and anatomic difference between mice and man? Is there a room of innovation by studying fungal microenvironment with simpler more experimentally controlled systems? (e.g. organoids)

REPLY : We agree that, although the currently available in vitro and in vivo models for preclinical medical mycology research are relevant for the development of antifungal therapies, immunological, pharmacological and anatomical differences between these models and humans remain a major source of bias in cell culture and animal experimentation. We also mentioned that studying the fungal microenvironment and evaluating new potent molecules/combinations using simpler, more experimentally controlled systems based on microfluidics or organoid design will certainly lead to promising new therapies in the near future. Finally, some relevant references were added (129-135) : **« Finally, although the currently available in vitro and in vivo models for preclinical medical mycology research are relevant for antifungal therapeutic development, the immunological, pharmacological, and anatomical differences between these models and humans remain a major source of bias in cell culture and animal experimentation (Curtis et al, 2025; Last et al, 2021). Studying the fungal microenvironment and evaluating new potent molecules/combinations using simpler, more experimentally controlled systems based on microfluidics or organoid design will certainly offer promising therapeutic avenues for innovation soon (Harding et al, 2025; Wang et al, 2021; Kim et al, 2021). »**

J) Is the way we measure susceptibility/resistance in vitro physiologically plausible to the in vivo situation? Is there room for innovation here? (e.g. studying different media in terms of iron, glucose sources, 02 tension) –

REPLY : Similarly, we think it is worth mentioning that the current methods used to measure the in vitro activity of antifungal compounds are physiologically implausible *in vivo*. Therefore, there is room for innovation in this field, for example in developing new synthetic media that mimic specific physiological conditions and microenvironments. We have now included a couple of sentences dealing with this, along with two recent references (lines 135-138) : **« Similarly, current methods used to measure the in vitro activity of antifungal compounds are physiologically implausible *in vivo*. Therefore, there is room for innovation in this field, for example, in the development of new synthetic media that mimic specific physiological conditions and microenvironments (Vahedi-Shahandashti et al, 2025; Ruhluel et al, 2022). »**

**Referee #2 (Remarks for Author):**

This is a very nice overview on the innovation of antifungal therapies. The authors have chosen to talk about both pathogen- and host-driven therapies. The paper is very well structured and written, giving the reader an immersive overview on the gaps and developments of much needed antifungal therapy. I just have a few of suggestions to improve an already excellent manuscript:

REPLY : This positive comment on our manuscript is appreciated.

1- Line 52 - remove "the" climate changes

REPLY : Correction done.

2- Line 53 - examples of thermotolerant species.

REPLY : We have now indicated *Candida auris* and *Coccidioides sp*. as thermotholerant species as follows (lines 53-54): **« Additionally, climate changes we are witnessing may have caused a global shift in the epidemiology of fungal infections, notably with the emergence and spread of thermotolerant species (*e.g. Candida auris, Coccidioides sp.*) »**

3- Line 138 - ROS are not exclusively targeting hyphal growth.

REPLY : We agree that ROS are not specific to hyphal forms and contribute broadly to antifungal activity against different fungal morphotypes. Our initial wording was imprecise; the reference to hyphal targeting was actually intended to apply to NETs). We have revised the sentence to distinguish clearly between the respective roles of ROS and NETs (line 155-157) : **« Neutrophils generate reactive oxygen species (ROS), which contribute broadly to antifungal activity, and can also release neutrophil extracellular traps (NETs), which are particularly effective against the hyphal fungal morphotype. »**

13th Apr 2026

Dear Prof. Papon,

We are pleased to inform you that your manuscript is accepted for publication and will be sent to our publisher to be included in the next available issue of EMBO Molecular Medicine pending the final figure adjustments by our graphic designer, who will contact you for your approval of the figures.

Your manuscript will be processed for publication by EMBO Press. It will be copy edited and you will receive page proofs prior to publication. Please note that you will be contacted by Springer Nature Author Services to complete licensing information.

Should you be planning a Press Release on your article, please get in contact with embo_production@springernature.com as early as possible in order to coordinate publication and release dates.

If you have any questions, please do not hesitate to contact the Editorial Office. Thank you for your contribution to EMBO Molecular Medicine.

Yours sincerely,
Zeljko Durdevic

Zeljko Durdevic
Senior Editor
EMBO Molecular Medicine

------------------------------------------------
